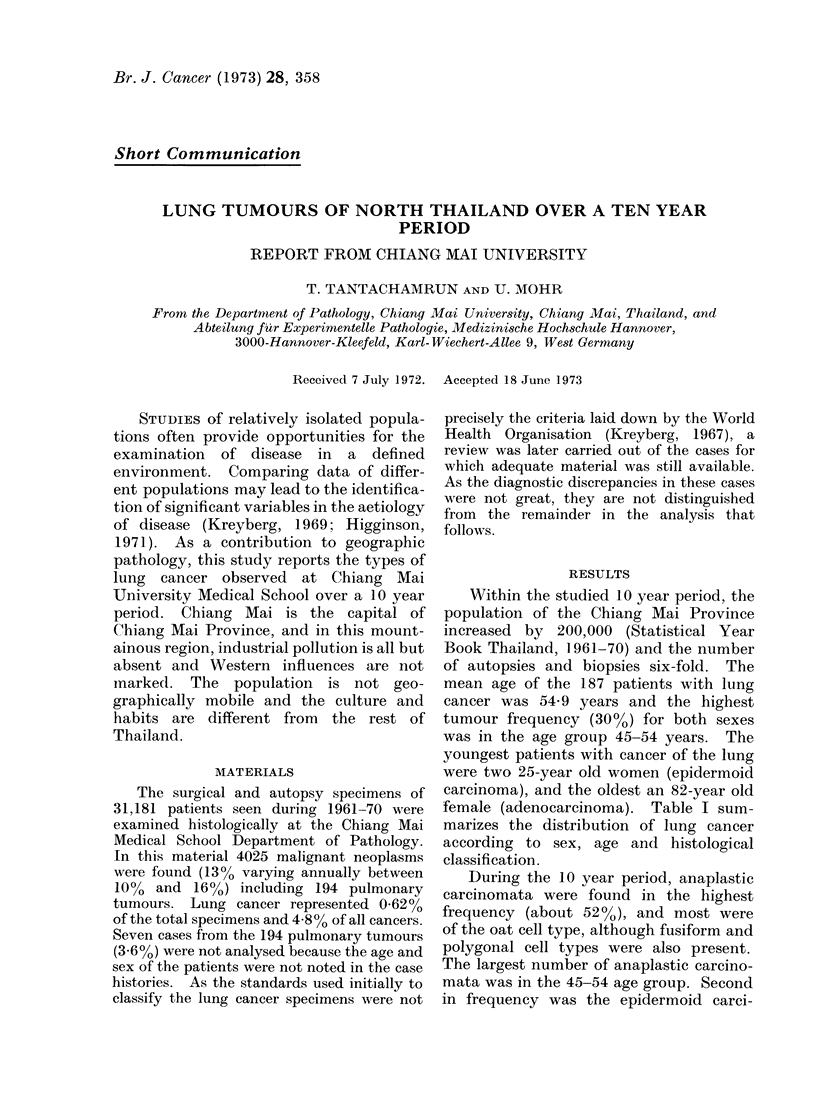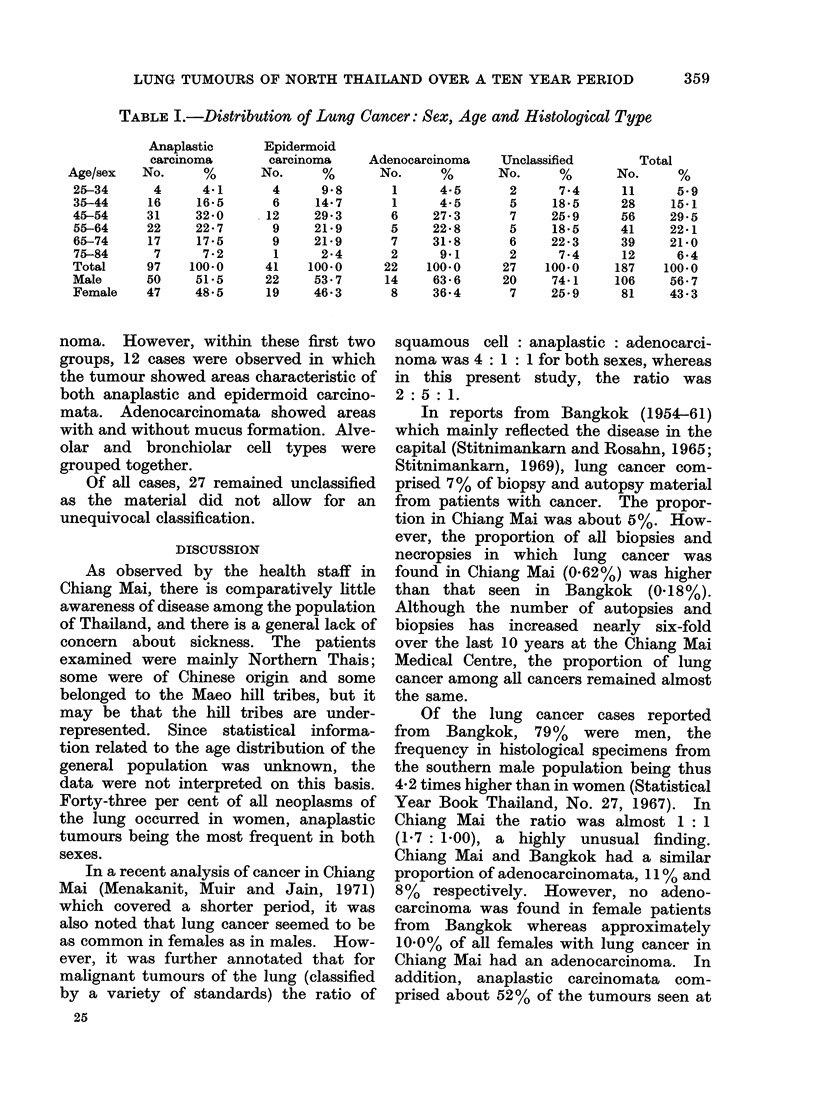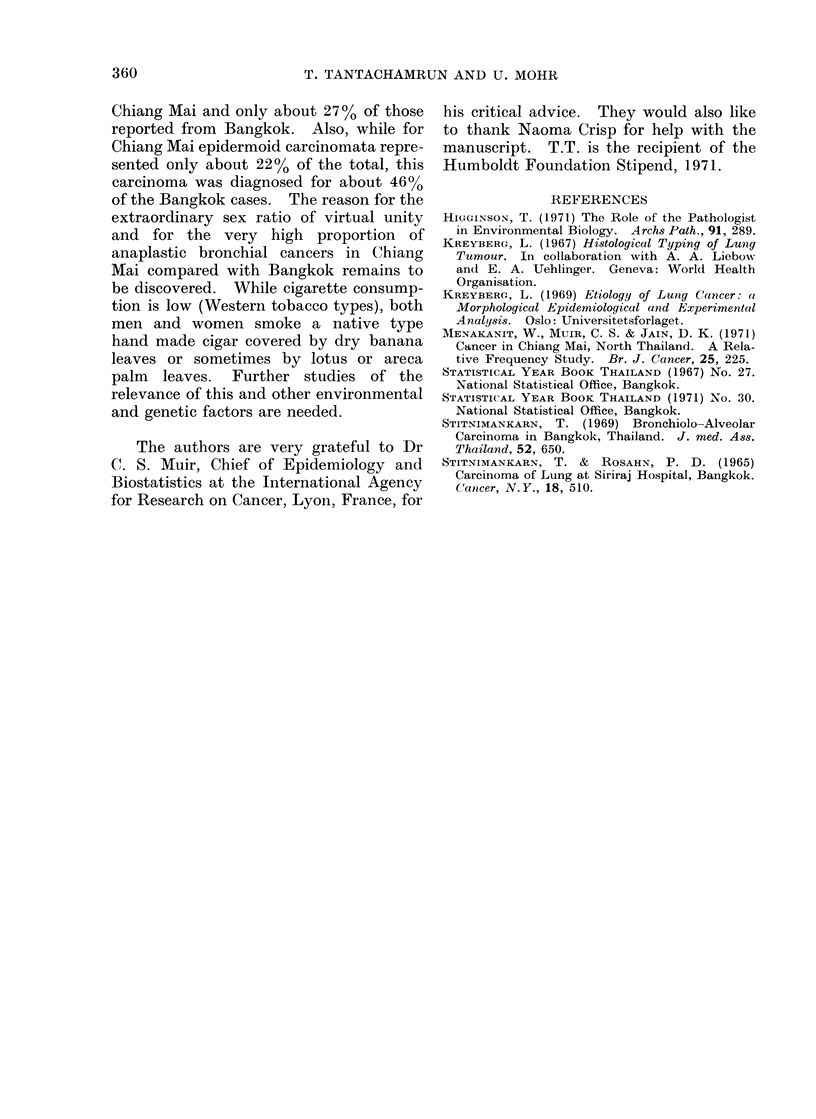# Lung tumours of north Thailand over a ten year period. Report from Chiang Mai University.

**DOI:** 10.1038/bjc.1973.160

**Published:** 1973-10

**Authors:** T. Tantachamrun, U. Mohr


*Br. J. Cancer* (1973) **28**, 358

***Short Communication***

### LUNG TUMOURS OF NORTH THAILAND OVER A TEN YEAR PERIOD

### REPORT FROM CHIANG MAI UNIVERSITY

T. TANTACHAMRUN AND U. MOHR

*From the Department of Pathology, Chiang Mai University, Chiang Mai, Thailand, and Abteilung für Experimentelle Pathologie, Medizinische Hochschule Hannover, 3000-Hannover-Kleefeld, Karl-Wiechert-Allee 9, West Germany*

Received 7 July 1972. Accepted 18 June 1973

STUDIES of relatively isolated populations often provide opportunities for the examination of disease in a defined environment. Comparing data of different populations may lead to the identification of significant variables in the aetiology of disease (Kreyberg, 1969; Higginson, 1971). As a contribution to geographic pathology, this study reports the types of lung cancer observed at Chiang Mai University Medical School over a 10 year period. Chiang Mai is the capital of Chiang Mai Province, and in this mountainous region, industrial pollution is all but absent and Western influences are not marked. The population is not geographically mobile and the culture and habits are different from the rest of Thailand.

### MATERIALS

The surgical and autopsy specimens of 31,181 patients seen during 1961–70 were examined histologically at the Chiang Mai Medical School Department of Pathology. In this material 4025 malignant neoplasms were found (13% varying annually between 10% and 16%) including 194 pulmonary tumours. Lung cancer represented 0·62% of the total specimens and 4·8% of all cancers. Seven cases from the 194 pulmonary tumours (3·6%) were not analysed because the age and sex of the patients were not noted in the case histories. As the standards used initially to classify the lung cancer specimens were not precisely the criteria laid down by the World Health Organisation (Kreyberg, 1967), a review was later carried out of the cases for which adequate material was still available. As the diagnostic discrepancies in these cases were not great, they are not distinguished from the remainder in the analysis that follows.

### RESULTS

Within the studied 10 year period, the population of the Chiang Mai Province increased by 200,000 (Statistical Year Book Thailand, 1961–70) and the number of autopsies and biopsies six-fold. The mean age of the 187 patients with lung cancer was 54·9 years and the highest tumour frequency (30%) for both sexes was in the age group 45–54 years. The youngest patients with cancer of the lung were two 25-year old women (epidermoid carcinoma), and the oldest an 82-year old female (adenocarcinoma). Table I summarizes the distribution of lung cancer according to sex, age and histological classification.

During the 10 year period, anaplastic carcinomata were found in the highest frequency (about 52%), and most were of the oat cell type, although fusiform and polygonal cell types were also present. The largest number of anaplastic carcinomata was in the 45–54 age group. Second in frequency was the epidermoid carci-

TABLE I.—*Distribution of Lung Cancer: Sex, Age and Histological Type*

| Age/sex | Anaplastic carcinoma No. | % | Epidermoid carcinoma No. | % | Adenocarcinoma No. | % | Unclassified No. | % | Total No. | % |
|---|---|---|---|---|---|---|---|---|---|---|
| 25–34 | 4 | 4·1 | 4 | 9·8 | 1 | 4·5 | 2 | 7·4 | 11 | 5·9 |
| 35–44 | 16 | 16·5 | 6 | 14·7 | 1 | 4·5 | 5 | 18·5 | 28 | 15·1 |
| 45–54 | 31 | 32·0 | 12 | 29·3 | 6 | 27·3 | 7 | 25·9 | 56 | 29·5 |
| 55–64 | 22 | 22·7 | 9 | 21·9 | 5 | 22·8 | 5 | 18·5 | 41 | 22·1 |
| 65–74 | 17 | 17·5 | 9 | 21·9 | 7 | 31·8 | 6 | 22·3 | 39 | 21·0 |
| 75–84 | 7 | 7·2 | 1 | 2·4 | 2 | 9·1 | 2 | 7·4 | 12 | 6·4 |
| Total | 97 | 100·0 | 41 | 100·0 | 22 | 100·0 | 27 | 100·0 | 187 | 100·0 |
| Male | 50 | 51·5 | 22 | 53·7 | 14 | 63·6 | 20 | 74·1 | 106 | 56·7 |
| Female | 47 | 48·5 | 19 | 46·3 | 8 | 36·4 | 7 | 25·9 | 81 | 43·3 |

noma. However, within these first two groups, 12 cases were observed in which the tumour showed areas characteristic of both anaplastic and epidermoid carcinomata. Adenocarcinomata showed areas with and without mucus formation. Alveolar and bronchiolar cell types were grouped together.

Of all cases, 27 remained unclassified as the material did not allow for an unequivocal classification.

### DISCUSSION

As observed by the health staff in Chiang Mai, there is comparatively little awareness of disease among the population of Thailand, and there is a general lack of concern about sickness. The patients examined were mainly Northern Thais; some were of Chinese origin and some belonged to the Maeo hill tribes, but it may be that the hill tribes are under-represented. Since statistical information related to the age distribution of the general population was unknown, the data were not interpreted on this basis. Forty-three per cent of all neoplasms of the lung occurred in women, anaplastic tumours being the most frequent in both sexes.

In a recent analysis of cancer in Chiang Mai (Menakanit, Muir and Jain, 1971) which covered a shorter period, it was also noted that lung cancer seemed to be as common in females as in males. However, it was further annotated that for malignant tumours of the lung (classified by a variety of standards) the ratio of squamous cell : anaplastic : adenocarcinoma was 4 : 1 : 1 for both sexes, whereas in this present study, the ratio was 2 : 5 : 1.

In reports from Bangkok (1954–61) which mainly reflected the disease in the capital (Stitnimankarn and Rosahn, 1965; Stitnimankarn, 1969), lung cancer comprised 7% of biopsy and autopsy material from patients with cancer. The proportion in Chiang Mai was about 5%. However, the proportion of all biopsies and necropsies in which lung cancer was found in Chiang Mai (0·62%) was higher than that seen in Bangkok (0·18%). Although the number of autopsies and biopsies has increased nearly six-fold over the last 10 years at the Chiang Mai Medical Centre, the proportion of lung cancer among all cancers remained almost the same.

Of the lung cancer cases reported from Bangkok, 79% were men, the frequency in histological specimens from the southern male population being thus 4·2 times higher than in women (Statistical Year Book Thailand, No. 27, 1967). In Chiang Mai the ratio was almost 1 : 1 (1·7 : 1·00), a highly unusual finding. Chiang Mai and Bangkok had a similar proportion of adenocarcinomata, 11% and 8% respectively. However, no adenocarcinoma was found in female patients from Bangkok whereas approximately 10·0% of all females with lung cancer in Chiang Mai had an adenocarcinoma. In addition, anaplastic carcinomata comprised about 52% of the tumours seen at

Chiang Mai and only about 27% of those reported from Bangkok. Also, while for Chiang Mai epidermoid carcinomata represented only about 22% of the total, this carcinoma was diagnosed for about 46% of the Bangkok cases. The reason for the extraordinary sex ratio of virtual unity and for the very high proportion of anaplastic bronchial cancers in Chiang Mai compared with Bangkok remains to be discovered. While cigarette consumption is low (Western tobacco types), both men and women smoke a native type hand made cigar covered by dry banana leaves or sometimes by lotus or areca palm leaves. Further studies of the relevance of this and other environmental and genetic factors are needed.

The authors are very grateful to Dr C. S. Muir, Chief of Epidemiology and Biostatistics at the International Agency for Research on Cancer, Lyon, France, for his critical advice. They would also like to thank Naoma Crisp for help with the manuscript. T.T. is the recipient of the Humboldt Foundation Stipend, 1971.

### REFERENCES

HIGGINSON, T. (1971) The Role of the Pathologist in Environmental Biology. *Archs Path.*, **91**, 289.

KREYBERG, L. (1967) *Histological Typing of Lung Tumour.* In collaboration with A. A. Liebow and E. A. Uehlinger. Geneva: World Health Organisation.

KREYBERG, L. (1969) *Etiology of Lung Cancer: a Morphological Epidemiological and Experimental Analysis.* Oslo: Universitetsforlaget.

MENAKANIT, W., MUIR, C. S. & JAIN, D. K. (1971) Cancer in Chiang Mai, North Thailand. A Relative Frequency Study. *Br. J. Cancer*, **25**, 225.

STATISTICAL YEAR BOOK THAILAND (1967) No. 27. National Statistical Office, Bangkok.

STATISTICAL YEAR BOOK THAILAND (1971) No. 30. National Statistical Office, Bangkok.

STITNIMANKARN, T. (1969) Bronchiolo–Alveolar Carcinoma in Bangkok, Thailand. *J. med. Ass. Thailand*, **52**, 650.

STITNIMANKARN, T. & ROSAHN, P. D. (1965) Carcinoma of Lung at Siriraj Hospital, Bangkok. *Cancer, N.Y.*, **18**, 510.